# Endometrial Stem Cells and Reproduction

**DOI:** 10.1155/2012/851367

**Published:** 2012-01-12

**Authors:** Sara S. Morelli, Pauline Yi, Laura T. Goldsmith

**Affiliations:** Department of Obstetrics, Gynecology and Women's Health, New Jersey Medical School, University of Medicine and Dentistry of New Jersey, Newark, NJ 07103, USA

## Abstract

Abnormal endometrial function remains a significant cause of implantation failure, recurrent pregnancy loss, and other pathologies responsible for female infertility. The development of novel therapies to treat infertility due to endometrial dysfunction requires an understanding of the latest advancements in endometrial cell biology, such as the role of endometrial stem cells. The remarkable regenerative capacity of the human endometrium is absolutely essential for successful reproduction and likely requires a population of stem cells in the endometrium. The purpose of this review is to provide an introduction to some of the newest concepts in endometrial stem cell biology.

## 1. Introduction

Successful reproduction in mammalian females requires a precisely timed and complex interaction between the hypothalamic-pituitary-ovarian (HPO) axis and the uterine endometrium. Although significant advances in reproductive medicine have achieved effective therapies for abnormalities in the HPO axis, abnormalities in endometrial function remain poorly understood. Abnormal endometrial function remains a significant cause of implantation failure, recurrent pregnancy loss, and other pathologies which lead to female infertility. Development of effective therapy for infertility due to endometrial dysfunction requires enhanced understanding of the latest advancements in endometrial cell biology such as the role of stem cells. The purpose of this review is to provide an introductory guide to understanding some of the newest concepts in endometrial stem cell biology.

The human endometrium, derived from the mucosal lining of the fused mesodermal (paramesonephric) tubes (the müllerian ducts) during embryogenesis, is a dynamic tissue. It is comprised of two major zones: (1) the functionalis, a transient layer containing glands extending from the surface (luminal) epithelium as well as the supportive stroma, and (2) the basalis, comprised of the basal region of the glands, stroma, supporting vasculature, and lymphoid aggregates. Although various leukocyte cell types populate the endometrial stroma, including T and B cells, mast cells, macrophages, and neutrophils, the majority of the leukocytes populating the decidualized endometrium of the late secretory phase and early pregnancy are phenotypically unique, tissue-specific lymphocytes known as uterine natural killer (uNK) cells [1]. These cells play a key role in the establishment and maintenance of early pregnancy [2]. Yet, despite their well-documented importance to successful reproduction, their lineage and origin remain unknown.

Human endometrium is unique in its temporally regulated processes of cellular proliferation, differentiation, and shedding of the functionalis layer with each menstrual cycle. The endometrium has the distinctive ability to undergo physiologic angiogenesis in order to facilitate implantation, as well as to regenerate an entirely new functionalis layer following each menses. This remarkable regenerative capacity is essential for successful human reproduction. Although the mechanisms which allow for it remain poorly understood, it is likely to require a uterine stem cell population [3–6].

Somatic stem cells have been identified in many tissue types, including intestine, skin, and bone marrow, and are crucial for physiologic tissue renewal and regeneration after injury [7]. Somatic stem cells are undifferentiated cells, defined by their ability to both self-renew and differentiate into mature progeny cells of a given tissue type. Evidence exists to support the presence of a resident stem cell population in the uterus, but the location and origin of these cells is unknown [3–6, 8, 9]. A number of possibilities exist as to the origin of endometrial stem cells [4, 6, 9]: (1) they may represent fetal epithelial and mesenchymal stem cells which remain in the adult endometrium and continue to replicate in adulthood, (2) they may represent circulating stem cells arising from a hematogenous source (such as the bone marrow) that seeds the endometrium either periodically or in response to injury, or (3) they may represent a combination of the above.

 The hypothesis that cyclic regeneration of the endometrium is mediated via a resident stem/progenitor cell population in the uterus was originally based on early experimental studies in rhesus monkeys, which revealed that removal of all visible tissue via endometriectomy was followed by, after a short delay, reconstruction of a new endometrium [10]. Clinical observations that some women who underwent complete endometrial ablation later developed areas of functional endometrial tissue [11] further supported this hypothesis. Subsequent studies by Padykula et al. [12, 13] provided additional, albeit indirect, evidence of the existence of an endometrial stem cell compartment. Using [^3^H]thymidine incorporation during the natural menstrual cycle of the rhesus monkey, these investigators demonstrated that the primate endometrium possessed a germinal compartment localized to the lower basalis, in which high epithelial activity persisted postovulation and appeared to escape inhibition by progesterone. This compartment persisted after menses and was postulated to give rise to the transient functionalis layer. These studies, performed in a primate model with menstrual cycles identical to that of the human, provided the basis for the hypothesis that the basalis is the location of a stem cell population in the human endometrium [12, 13].

## 2. Endometrial Stem Cells: Evidence for Their Existence

Adult stem cells in the endometrium are difficult to identify because they constitute very small populations of cells, and because cell surface markers specific for adult stem cells have not been definitively characterized [4]. Studies which provide indirect evidence for the existence of endometrial stem cells do so by characterizing cell populations in the endometrium which exhibit the functional properties of stem cells. These properties include clonogenicity, proliferative potential, and capacity for differentiation into one or more lineages [4]. Such functional assays provide evidence for the existence of adult stem cells but unfortunately do not allow for localization of the cells within a given tissue.

 Clonogenicity, defined as the ability of a single cell to produce a colony when seeded at very low densities, was demonstrated in human endometrium for the first time in 2004 [14]. Using purified single cell suspensions dispersed from hysterectomy specimens, Chan et al. identified small populations of epithelial (0.22%) and stromal cells (1.25%) in human endometrium that possessed clonogenic activity [14]. Large colonies containing >4000 cells were rare and postulated to be initiated by stem/progenitor cells, whereas the more common small colonies were postulated to be initiated by more mature transit amplifying cells. A more recent study by these investigators [15] demonstrated that, for both epithelial and stromal cells, clonogenicity did not vary by cycle phase or between active (cycling) and inactive endometrium. The finding, however, of clonogenic cells in inactive endometrium further supports the existence of an endometrial stem cell niche in the basalis, as inactive endometrium is predominantly basalis and lacks functionalis [15].

Other properties evaluated in characterization of an endometrial stem cell population include the capacity for uni- or multilineage differentiation. The differentiation potential of candidate stem cells is evaluated after culturing the cells in differentiation-induction media, then analyzing the cells for expression of phenotypic differentiation markers. Gargett et al. [16] evaluated proliferative and differentiation potential of clonogenic human endometrial cells. Proliferative potential was assessed by serially passaging individual epithelial and stromal colony forming units (CFU) until senescence; large CFU underwent >30 population doublings before senescence, indicating their high proliferative potential characteristic of stem/progenitor cells. Single epithelial CFU differentiated into mature glands in vitro, and large secondary stromal clones demonstrated multipotency as their progeny differentiated into smooth muscle cells, adipocytes, chondrocytes, and osteoblasts, when cultured in typical differentiation-induction media. Thus, both epithelial progenitor cell and multipotent mesenchymal stem cell- (MSC-) like populations were identified in human endometrium. MSCs are multipotent cells located in the bone marrow and multiple other tissues and have the ability to differentiate into cells of multiple mesoderm-derived lineages, such as bone, cartilage, muscle, and adipose tissue [17]. Similarly, Wolff et al. [18] demonstrated the presence of multipotent cells in human endometrium by inducing chondrogenic differentiation of a subpopulation of endometrial stromal cells in vitro, identified by expression of type II collagen and sulfated glycosaminoglycans, characteristic of chondrocytes. It must be recognized, however, that although these studies provide evidence for the existence of putative endometrial epithelial and stromal stem cells, differentiation studies do not rule out the possibility of dedifferentiation of mature stromal cells in the presence of differentiation-induction media, a major limitation of the existing human in vitro differentiation studies.

Another approach used by multiple investigators to identify and characterize stem cells in the human endometrium is the isolation of cells with the “side population” phenotype. Side population cells are characterized by their ability to exclude the DNA-binding dye Hoechst 33343 by expressing ATP-binding cassette transporter proteins [19] and exhibit the properties of adult stem cells including long-term proliferative potential and differentiation into mature tissue-specific cell types. This method has been used to identify putative stem cell populations in multiple tissues, including bone marrow [19], liver [20], mammary gland [21], skin [22], and kidney [23]. More recently, this method has been utilized for the identification and characterization of stem cells in human endometrium. Side population cells isolated from human endometrium have been demonstrated to display long-term proliferative properties as well as differentiation into mature endometrial glandular epithelial, stromal, and endothelial cells both in vitro [24, 25] and in vivo in immunodeficient mouse models [25–27]. Additional studies have reported the ability of endometrial side population cells to differentiate in vitro into adipocytes and osteocytes, supporting a mesenchymal origin of these cells [26, 28]. Taken together, the growing amount of literature utilizing this technique, albeit limited to few laboratories worldwide, supports the hypothesis that side population cells isolated from human endometrium are indeed somatic stem cells, and that these cells are a source of mature endometrial cell types.

Although phenotypic markers specific to endometrial stem cells have yet to be definitively identified, Schwab and Gargett [29] demonstrated that the perivascular markers CD146 and PDGF-R*β* enabled isolation of stromal cells from human endometrium which exhibit phenotypic and functional properties of mesenchymal stem cells (MSCs). The investigators then used immunohistochemistry to localize these cells to perivascular areas of the basalis and functionalis. They hypothesized that these endometrial “MSC-like” cells may contribute to cyclic regeneration of the endometrium and further postulated that they may play a role in the pathogenesis of diseases such as endometriosis and adenomyosis [29].

## 3. Localization of Endometrial Stem Cells: Mouse Models

A major limitation of the human studies performed to date is their inability to definitively identify the origin and location of candidate stem cells in the endometrium. Investigators have thus turned to mouse models, using a technique which takes advantage of the quiescent nature of stem cells. In this approach, known as the label-retaining cell (LRC) approach, animals are injected with a thymidine analogue (bromodeoxyuridine or BrdU) which becomes incorporated into genomic DNA during the replication phase of mitosis, and the tissue of interest is examined for cells which retain this label after a prolonged chase period due to infrequent cell divisions (characteristic of somatic stem cells). Using this technique, Chan and Gargett [30] identified 3% of epithelial cells (predominantly luminal) and 6% of stromal cells which were adjacent to the luminal epithelium at the endometrial-myometrial junction, as LRC. A subsequent study [31] detected stromal LRC in a similar location, but none in the epithelial compartment, after a prolonged chase period. A more recent study [32] did not evaluate the stromal compartment but identified epithelial LRCs predominantly in the glandular epithelium. Thus, the existing data on the location of the endometrial stem cell niche in a mouse model are unclear as to cell type(s) and require further study, but they support the existence of a small population of uterine stem cells which are a likely source of regenerative endometrium.

## 4. The Bone Marrow as a Source of Endometrial Stem Cells

Mesenchymal stem cells (MSCs) from the bone marrow have been demonstrated to differentiate into mature cell types of various nonhematopoietic organs including liver, skeletal muscle, brain, and skin [33]. Recent data from a limited number of investigators support the concept that bone marrow is an important contributor of stem cells to the endometrium. Three independent investigators have identified human endometrial stromal, glandular, and/or endothelial cells of donor bone marrow origin in a total of 8 recipients of bone marrow transplantation from either HLA-mismatched [34] or male [35, 36] donors. These findings were provocative, supporting the ability of bone marrow-derived cells to generate endometrial cells de novo. To date, only three independent laboratories have reported the use of a murine bone marrow transplant model to determine whether bone marrow-derived cells give rise to endometrial parenchymal cell types. Du and Taylor [37] identified Y chromosome-positive endometrial epithelial and stromal cells in female recipients of bone marrow transplant from male donors six months post transplant. A similar approach was used by Mints and colleagues [36], who performed murine bone marrow transplantation using male donors and identified endothelial cells of donor origin in recipient endometria 40 days post transplant. Bratincsák et al. [38] created a transgenic mouse model in which all CD45^+^ cells coexpressed Green Fluorescent Protein and demonstrated that CD45^+^ hematopoietic progenitor cells contributed to uterine epithelium. Collectively, these data, albeit limited, support the hypothesis that the bone marrow is an important source of endometrial stem cells, exhibiting the capacity to differentiate into parenchymal and endothelial cell types.

Only one laboratory to date has previously tested the in vitro capacity of human bone marrow-derived cells to differentiate into mature endometrium. Multipotent mesenchymal stem-like cells which express cell surface markers typical of bone marrow MSCs have been identified in human endometrium [16, 29]. Given this line of evidence, Aghajanova et al. [39] recently tested the ability of human bone marrow-derived MSCs to differentiate into endometrial decidual cells. Human neonatal dermal fibroblasts were used as a control to determine whether mature fibroblasts could transdifferentiate under the same culture conditions. After 14 days of treatment with 8-bromo-cyclic adenosine monophosphate (a potent decidualizing agent of human endometrial stromal cells), human bone marrow-derived MSCs (but not dermal fibroblasts) displayed morphologic features characteristic of decidual cells and expressed the classical markers of decidualization, prolactin and IGFBP-1. These studies further support the bone marrow as a potential precursor of endometrial cells. Whether another major population of bone marrow-derived cells, lymphohematopoietic stem cells (precursors of all hematopoietic lineages), are a potential progenitor of human endometrial parenchymal or immune cell types remains to be investigated. Nonetheless, the concept of bone marrow-derived endometrial progenitor cells is a provocative one and bears significance not only in mechanisms underlying normal endometrial physiology but also in disorders of endometrial proliferation, such as endometriosis, endometrial hyperplasia, and endometrial carcinoma. Resident (as opposed to bone marrow-derived) epithelial and/or “MSC-like” stem cells may also contribute to such diseases [4, 6, 8, 40]. Furthermore, the bone marrow as a source of endometrial cells has therapeutic implications in the treatment of diseases such as Asherman's syndrome, or poorly understood disorders of implantation, important causes of female infertility.

## 5. Uterine Natural Killer (uNK) Cells

Another major limitation of the uterine stem cell studies performed to date is that the cellular sources of endometrial epithelium and stroma have been the predominant focus of study, with limited attention to the cellular origin of a critical cell type in endometrial function: the uterine natural killer (uNK) cell. Phenotypically and functionally different from peripheral NK cells, these cells are crucial in the establishment and maintenance of early pregnancy [2]. They become abundant in the human uterus 3–5 days post ovulation and by late secretory phase account for at least 30% of the endometrial stroma [41]. Human in vitro studies provide evidence for the ability of uNK cells to promote placental vascular growth and decidualization via production of chemokines and angiogenic factors [2]. Similarly, murine studies indicate that uNK cells are essential for induction of spiral arteries, mediated via their production of IFN*γ* [42]. Indeed, the finding that uNK-deficient mice exhibit compromised placentation and fetal growth [43] supports a critical role of the uNK cell in reproductive function. However, despite their importance in reproductive function, the cellular origin of uNK cells remains unclear.

A small number of studies have been performed utilizing murine bone marrow transplant models and human in vitro studies, which support the bone marrow as a source of uNK cells. Peel et al. [44], who performed some of the earliest studies which provided evidence for bone marrow origin of uNK cells, utilized a rat-to-mouse bone marrow transplant model to identify donor-derived uNK cells in deciduomata of pseudopregnant recipients. Guimond et al. [45] subsequently demonstrated in murine studies that bone marrow transplantation from NK cell-competent to NK cell-deficient mice led to restoration of the uNK cell population in recipients as well as restoration of normal decidualization/placentation and fetal viability. Further evidence for a nonuterine source of uNK cells has been provided by Chantakru et al. [46], who demonstrated that grafting of uterine segments from NK cell-competent mice into uNK cell-deficient mice revealed the absence of uNK cells in the decidualized grafts. However, transfer of cells from secondary lymphoid tissues (thymus, spleen, peripheral and mesenteric lymph nodes) reconstituted the uNK cell population in recipients. More recently, Vacca et al. [47] demonstrated the ability of CD34^+^ cells (the phenotype of bone marrow-derived hematopoietic precursors) present in human decidua to differentiate in vitro into uNK cells, either in the presence of certain growth factors or in coculture with decidual stromal cells. These uNK cells were functional (producing IL-8 and IL-22, characteristic products of human uNK cells) and expressed phenotypic cell surface markers of human uNK cells (CD56^bright^/CD16^−^). However, additional studies, particularly in human tissues, are clearly necessary in order to clarify whether or not the bone marrow represents a significant source of uNK cell precursors.

In summary, additional studies are necessary to provide us with an enhanced understanding of the capabilities and limitations of endometrial stem cells to determine the potential therapeutic uses for these cells.
